# Immunodiagnostic Significance of Anti-RA33 Autoantibodies in Saudi Patients with Rheumatoid Arthritis

**DOI:** 10.1155/2015/604305

**Published:** 2015-03-25

**Authors:** Jamil A. Al-Mughales

**Affiliations:** Department of Clinical Laboratory Medicine (Diagnostic Immunology Division) and Department of Medical Microbiology and Immunology, Faculty of Medicine, King Abdulaziz University, P.O. Box 80215, Jeddah 21519, Saudi Arabia

## Abstract

The primary objective of this study was to evaluate and compare the immunodiagnostic significance and utility of anti-RA33 with anti-CCP, RF, and CRP in Saudi patients with rheumatoid arthritis. *Methods*. This was a prospective controlled clinical study conducted at King Abdul Aziz University Tertiary Medical Centre. The sera of 41 RA patients, 31 non-RA patients, and 29 healthy controls were collected. Anti-RA33 and anti-CCP were measured using commercially available ELISA principle kits. RF and CRP were measured using nephelometry. *Results*. Anti-RA33 antibodies had the lowest positive and negative predictive values and showed a sensitivity of 7.32% with 95.12% specificity. Of the other three markers (including anti-CCP antibodies, CRP, and RF), only anti-CCP showed specificity of 90.46% with sensitivity of 63.41% compared to non-RA patients + healthy control. There was a significant correlation with rheumatoid factor positivity with anti-CCP. With respect to CRP, a notable correlation was seen only with anti-RA33. *Conclusion*. Compared to rheumatoid factor, anti-CCP antibodies, and C-reactive proteins, the anti-RA33 autoantibodies seem to be not representing as an important additional immunodiagnostic marker in Saudi patients with established RA. RA33 may have more interest in early RA or less severe RA and other systemic connective tissue disorders.

## 1. Introduction

The production of autoantibodies against specific or several self-antigens in the body is the diagnostic hallmark of autoimmune disorders. The cause of such antibody production remains unknown and inadequately explained [1–3]. Under autoimmune conditions, certain components of the human cell are specifically targeted by autoantibodies. For example, in systemic lupus erythematosus, autoantibodies are produced against DNA (anti-dsDNA); in mixed connective tissue disease, autoantibodies are produced against ribonuclear protein (anti-RNP); in progressive systemic sclerosis, autoantibodies are produced against anti-t-RNA synthetase (anti-Scl70); and in polymyositis-dermatomyositis, autoantibodies against RNA are synthesized, anti-JO-I [4].

In the 1940s, the concept of autoimmunity in RA was proposed by Waaler, who threw light on the disturbances in the connective tissue metabolism involved in this disease [5]. Waaler demonstrated that the autoantibody RF is elevated in RA patients. Several years later, in 1970, Steffen hypothesized that RA could be a collagen autoimmune disease [6]. Subsequently, animal studies on type II collagen-induced arthritis confirmed this hypothesis [7–10]. Past clinical evidence has demonstrated the production of several autoantibodies, including RF and other anti-collagen antibodies, in synovial plasma cells in response to RA pathogenesis. These findings suggest local antigen activity in the immune response activation in synovial tissue [11–13]. Hitherto, and despite its nonspecificity, RF is still widely employed in the diagnostic work-up for RA [14–16]. Further recent studies suggest that combined utilisation of IgM and IgA RF autoantibodies offers higher specificity in RA, in comparison with IgM RF alone [17].

However, there still was a need to explore other diagnostic markers with greater specificity for RA. Substantial research that has been conducted in this direction came up with newer interesting markers such as anti-MCV and, more recently, anti-CCP antibodies, which showed a satisfying specificity in immunodiagnosis of RA [18–25]. Other studies reported another immunodiagnostic marker in RA patients called anti-RA33 [26].

Anti-CCP antibodies were discovered while exploring the sera of RA patients for further autoantibodies distinct from RF and anti-MCV. The first citrullinated-binding autoantibodies in rheumatoid sera were discovered by Niehus and Mandema in 1964 [21]. These autoantibodies demonstrated the ability to bind to perinuclear granules in normal human buccal mucosal cells and were named antiperinuclear factor. Past studies also showed that these autoantibodies occur in 48% of RA patients and only in 1% of healthy controls [22]. Subsequent studies discovered that conversion of arginine to citrulline on peptides was essential for anti-keratin antibody and perinuclear factor binding. Hence, these autoantibodies were later called anti-citrullinated peptide antibodies. Recent immunodiagnostic advances have further subdivided anti-CCP into anti-CCP1, anti-CCP2, and anti-CCP3 [22, 23]. Anti-CCP has a sensitivity range of 39–89% and a specificity range of 50–99% for the diagnosis of RA [24, 25, 27–29].

Anti-RA33 autoantibody, directed to RA33 complex, or heterogeneous nuclear ribonucleic protein (hnRNP), has been identified in RA patients sera since 1989 [4, 26, 30]. However, hnRNP-A2 (and its alternatively spliced variants B1 and B2) is the sole epitope of RA33 that is reported to be potentially autoantigenic in RA patients. Actually, about 30 different epitopes of hnRNP have been discovered, each epitope referring to the specific protein sequence combined to pre-mRNA to form the hnRNP complex. Besides, hnRNP-A2, a few other members of the big family of hnRNPs, have shown autoantigenic involvement, with more or less affinity, in systemic autoimmune rheumatoid diseases [3, 31]. Accordingly, detection of anti-RA33 autoantibodies has emerged in rheumatology practice, especially anti-A2/hnRNP (commonly referred to as anti-RA33) as an additional immunodiagnostic marker for RA [3, 30].

However, the diagnostic utility of anti-RA33 autoantibody in RA is still controversial, as it was recognized in a low proportion of RA patients [26, 30, 32]. Further researchers have demonstrated that anti-RA33 shows reliable sensitivity and specificity in patients with established RA [33, 34].

On the other hand, introducing anti-RA33 in the list of RA diagnostic markers is certainly a noteworthy progress in the field of RA research, whose real utility needs to be further explored.

The current study investigates the diagnostic reliability of anti-RA33 as an additional marker for RA compared to current immunodiagnostic markers including anti-CCP, RF, and CRP, among Saudi patients attending the rheumatology clinic at King Abdulaziz University's Tertiary Care Medical Centre. This is the first time that such a study has been conducted in the Saudi population, which should further provide valuable data regarding the prevalence of RA33 autoantigen in this population. Moreover, although the reliability of these fairly autoantibodies has been reported before, the data is still scarce, and this study therefore contributes to and builds on the already accumulated evidence.

## 2. Materials and Methods

### 2.1. Patients

This was a controlled clinical study conceptualized, designed, and conducted at the Diagnostic Immunology Division of King Abdulaziz University Tertiary Medical Centre. A total of 41 sera of Saudi RA patients were enrolled in this study. All patients were having established RA and meeting ACR classification criteria diagnosed by rheumatologist according to revised American College of Rheumatology (ACR) criteria for RA [35].

A total of 31 non-RA patients (OA 7, SLE 5, SS 3, MCTD 3, other diseases 13) were also studied. They were assigned as controlled group. Inclusion criterion was RA which had been diagnosed by rheumatologist. An exclusion criterion was those suffering from connective tissue disorders. In addition, 29 healthy individuals were included as a healthy control group. All patients and controls are from the same ethnic origin.

C-reactive protein and RF levels were available for all patients.

The study was approved by the Ethics Committee at King Abdulaziz University Medical Centre.

### 2.2. Antibody Detection

Anti-CCP antibodies were detected using the commercially available Alegria instrument (Orgentic, Hamburg, Germany) according to the manufacturer's instructions. Anti-RA33 antibody was detected using a commercially available enzyme-linked immunosorbent assay (ELISA) kit (Human, Wiesbaden, Germany) according to the manufacturer's instructions. RF and CRP were also measured using a nephelometer (Siemens, Germany).

The assay reaction for anti-RA33 involves covalent immobilisation of recombinant RA33 (hnRNP/A2) to the solid phase of microtiter strips and subsequent binding of anti-RA33 antibodies from patient serum. The bound antibodies are detected with a peroxidase-labelled secondary antibody that is directed against human IgG. After addition of the substrate solution, the antibodies are stained. The intensity of the colour is proportional to the concentration and/or the avidity of the detected antibodies. After the addition of stop solution, the colour changes from blue to yellow. The results were calculated from the standard curve obtained.

The cut-off point is taken as recommended by the manufacturers (mentioned above) including anti-RA33 (above 25 U/mL are considered positive), anti-CCP (above 20 U/mL), CRP (above 3.5 mg/L), and RF (above 10 U/mL).

### 2.3. Statistical Analysis

The RA group was evaluated using descriptive statistics: the means of the continuous variables were calculated, and frequency percentages were calculated for all categorical variables. To determine the reliability of the diagnostic test, sensitivity, specificity, positive predictive value (PPV), and negative predictive value (NPV) were calculated. As the sample size was less than 50, the qualitative relationship of each antibody with CRP and RF was assessed using Fischer's exact chi-square test. Further, Pearson's correlation analysis was used to assess the quantitative relationship of the anti-RA33 and anti-CCP assays with CRP and RF. A linear regression model was built for analyzing the nature of the relationship between all four diagnostic tests and the dependent variables. All statistical analyses were conducted using the Statistical Package for Social Sciences (SPSS) version 19.1.

## 3. Results

### 3.1. Patient Characteristics

The disease showed a female predilection (80.5%) among the study of RA patients. The assay findings showed positive results for anti-RA33 (7.3%), anti-CCP (63.4%), and CRP (80.5%) and 51.2% were positive for rheumatoid factor.  Table 1 summarizes the demographic characteristics of the study groups (RA patients, non-RA patients and healthy controls); and Table 2 summarizes the results of the immunodiagnostic markers in the three study groups.

### 3.2. Sensitivity, Specificity, NPV, and PPV

The anti-CCP antibody showed 63.41% sensitivity for the detection of RA, and the anti-RA33 antibody showed a sensitivity of 7.32%. Anti-CCP demonstrated more favourable predictive values for RA than anti-RA33.  Table 3 summarizes the sensitivity, specificity, PPV, and NPV values for the markers investigated in the study.

### 3.3. Qualitative Analysis

Results of the qualitative analysis revealed that, compared to the anti-RA33 test, the anti-CCP test was significantly more efficacious (*P* = 0.004) in identifying 18 patients with rheumatoid factor positivity. The anti-RA33 results were not significant compared with rheumatoid factor (*P* = 0.232) (Table 4).

### 3.4. Quantitative Analysis

The results of the quantitative analysis revealed a strong positive correlation (*r* = 0.57) between the anti-RA33 and CRP values. However, a similar trend was not observed for the anti-RA33 and RF values. However, anti-CCP did not show any such correlation with CRP or RF.  Table 5 summarizes the results of the correlation analysis.

### 3.5. Linear Regression Analysis

A linear regression analysis was also performed to understand the association between the examined diagnostic markers of RA and the CRP and RF values. The linear regression model demonstrated that only the anti-RA33 values changed with respect to the CRP values in a statistically significant manner (*P* ≤ 0.001). No notable correlation was observed between anti-CCP and the CRP/RF values. The details of this analysis have been summarized in Table 6.

## 4. Discussion

In Saudi Arabia, there are no valuable reported evidence-based studies indicating the immunodiagnostic role of anti-RA33 in adult RA patients. The current study shows the evidence of inferior diagnostic value of anti-RA33, compared to anti-CCP, but also compared to CRP and RF in the immunodiagnosis of RA.

The reported association between anti-CCP and RA was confirmed in our study. Conversely, the values of sensitivity and specificity of anti-CCP test vary from one study to another. In a study by Kaptanoğlu et al. [36], the sensitivity and specificity were 53% and 79%, while in Awwad and Aboukhamis [32] they were reported to be 71.9% and 100%, respectively. Other studies also showed sensitivity range of 39–89% and a specificity of 50–99% for the diagnosis of RA [24, 25, 27–29].

However, the anti-CCP test values alone were significant in correctly identifying patients with RF positivity, as compared to the anti-RA33 test. On the other hand, changes in CRP values better correlate with the anti-RA33 values, which led us to infer that anti-CCP test could be used in identifying RF positive individuals. This might support utilizing this combination in monitoring the relapsing-remitting of the disease, which is compatible with previous studies that have confirmed that anti-CCP combined with RF appears to be even better prognostic marker [37].

In case of anti-RA33 antibodies, our study has indicated sensitivity of 7.3% and 96.5% specificity. Other authors reported 6–58% sensitivity and specificity of 69–96% [26, 29–31, 37, 38]. Although they do not mention the autoantigen source in their ELISA methods, few authors reported controversial data including 98% sensitivity and 20% specificity for anti-RA33 in RA patients [34]. However, our relative low sensitivity can be explained by the fact that the population of our study excluded early RA patients, as it concerned only established RA. Additionally, the significant linear relation between RA33 and CRP suggests that the few patients with positive RA33 have less severe RA.

In addition, to less sensitivity of anti-RA33, other previous studies confirm that anti-RA33 is not exclusively present in RA [4]. It is also present in SLE and MCTD [4]. Our study has observed only 1/5 SLE positive anti-RA33, but our sample size was not large enough to confirm the previous reported studies.

Although our findings were in agreement with most studies, the differences between our results and other studies reported above might be attributed to either RA severity or ethnic origin or might be due to the degree of the purification of the RA33 that has been used as recombinant autoantigens source in their ELISA methods. This is supported by recent data where authors used hnRNP B1 (RA33) as autoantigens and also suggested the influence of genetic involvement [31]. Moreover, the same authors reported that anti-hnRNP B1 autoantibodies are significantly more prevalent in RA patient with combined systemic sclerosis and hypertension [31].

In conclusion, our study suggests that anti-RA33 (IgG) autoantibodies (anti-hnRNP/A2) occur in Saudi RA patients with very low diagnostic sensitivity (7.32%), which seems to be not representing as an additional immunodiagnostic marker in established RA. In addition, it would be interesting to do larger future prospective studies to address the diagnostic significance of these autoantibodies in early RA and in established RA with less severe forms and in other connective tissue disorders.

## Figures and Tables

**Table 1:** Demographic characteristics of the study population.

Variables	Mean (SD) or frequency (%)		
	RA patients (*n* = 41)	Non-RA patients (*n* = 31)	Healthy controls (*n* = 29)
Age (years)	46 14.5	44 17.6	27.6 6.7
Sex			
Female	33 80.5	23 74.2	3 10.3
Male	8 19.5	8 25.8	26 89.6

**Table 2:** Diagnostic immunology markers in RA and non-RA patients and healthy controls.

Variables	Mean SD or frequency (%) of RA (*n* = 41)	Mean SD or frequency (%) of non-RA (*n* = 31)	Mean SD or frequency (%) of healthy controls (*n* = 29)
C-reactive protein			
Negative	8 19.5	18 58.1	22 75.9
Positive	33 80.5	13 41.9	7 24.1
Rheumatoid factor			
Negative	20 48.8	23 74.2	28 96.6
Positive	21 51.2	8 25.8	1 3.4
Anti-CCP antibody			
Negative	15 36.6	27 87.1	28 96.55
Positive	26 63.4	4 12.9	1 3.4
Anti-RA33 antibody			
Negative	38 92.7	30 96.7	28 96.6
Positive	3 7.3	1 3.2	1 3.4

**Table 3:** Predictive value, specificity, and sensitivity of the anti-CCP and anti-RA33 tests in RA diagnosis.

	Anti-CCP	Anti-RA33
Compared to healthy donors		
Sensitivity (%)	63.4	7.3
Specificity (%)	96.5	96.5
Positive predictive value (%)	96.3	75.0
Negative predictive value (%)	65.1	42.4
Compared to non-RA patients		
Specificity (%)	87.1	96.7
Positive predictive value (%)	86.6	75.0
Negative predictive value (%)	64.2	44.1
Compared to non-RA patients and healthy controls		
Specificity (%)	90.2	95.1
Positive predictive value (%)	86.6	60.0
Negative predictive value (%)	71.1	50.6

**Table 4:** Association of each of the autoantibodies with RF.

Variables	Rheumatoid factor		
	Negative	Positive	*P* value
Anti-CCP			
Negative	12	3	0.004^*^
Positive	8	18	
Anti-RA33			
Negative	20	18	0.232
Positive	0	3	

^*^significant result (*P* < 0.05).

**Table 5:** Correlation of the diagnostic markers of RA with CRP and RF values.

	C-reactive protein	*P* value	Rheumatoid factor	*P* value
Anti-CCP	−0.204	0.1	0.250	0.058
Anti-RA33	0.575	<0.001^*^	−0.006	0.5

^*^significant result (*P* < 0.05).

**Table 6:** Linear regression analysis of the diagnostic markers of RA with CRP and RF values.

Variables	C-reactive protein			Rheumatoid factor	
	Constant	*B*-coefficient	*P* value	*B*-coefficient	*P* value
Anti-CCP	328.3	−0.192	0.220	0.240	0.128
Anti-RA33	−8.47	0.576	<0.001^*^	0.024	0.857

Linear regression shows significant correlation between anti-RA33 values and CRP changes.

No notable correlation was observed between anti-CCP and the CRP or RF values.

^*^significant result (*P* < 0.05).

## Abbreviations

RA33: Nuclear autoantigen with an apparent molecular mass of 33 kd
Anti-CCP: Anti-citrullinated cyclic peptide
Anti-MCV: Anti-mutated citrullinated vimentin
RF: Rheumatoid factors
CRP: C-reactive protein
hnRNP: Heterogeneous nuclear ribonucleoprotein
ELISA: Enzyme Linked Immunosorbent Assay
SLE: Systemic lupus erythematous
SS: Sjorgren's syndrome
MCTD: Mixed connective tissue diseases
OA: Osteoarthritis
ACR: American College of Rheumatology.
